# Direct and Indirect endocrine-mediated suppression of human endometrial CD8+T cell cytotoxicity

**DOI:** 10.1038/s41598-021-81380-8

**Published:** 2021-01-19

**Authors:** Z. Shen, M. Rodriguez-Garcia, M. V. Patel, C. R. Wira

**Affiliations:** 1 Department of Microbiology and Immunology, Geisel School of Medicine at Dartmouth, One Medical Center Drive, Lebanon, NH 03756 USA; 2 Department of Immunology, Tufts University School of Medicine, Boston, MA USA

**Keywords:** Cytotoxic T cells, Mucosal immunology, Transforming growth factor beta

## Abstract

Regulation of endometrial (EM) CD8+T cells is essential for successful reproduction and protection against pathogens. Suppression of CD8+T cells is necessary for a tolerogenic environment that promotes implantation and pregnancy. However, the mechanisms regulating this process remain unclear. Sex hormones are known to control immune responses directly on immune cells and indirectly through the tissue environment. When the actions of estradiol (E_2_), progesterone (P) and TGFβ on EM CD8+T cells were evaluated, cytotoxic activity, perforin and granzymes were directly suppressed by E_2_ and TGFβ but not P. Moreover, incubation of polarized EM epithelial cells with P, but not E_2_, increased TGFβ secretion. These findings suggest that E_2_ acts directly on CD8+T cell to suppress cytotoxic activity while P acts indirectly through induction of TGFβ production. Understanding the mechanisms involved in regulating endometrial CD8+T cells is essential for optimizing reproductive success and developing protective strategies against genital infections and gynecological cancers.

## Introduction

The mucosal immune system in the human female reproductive tract (FRT) is precisely regulated to optimize conditions for successful fertilization, implantation, and pregnancy while providing protection against sexually transmitted infections (STIs)^1^. Unlike other sites in the FRT, the endometrium must protect itself from sexually transmitted pathogens while allowing for the presence of allogeneic spermatozoa and supporting an immunologically distinct fetus. Key to this unique balance within the FRT are the sex hormones, estradiol (E_2_) and progesterone (P), which are secreted by the ovary and whose levels fluctuate during the menstrual cycle and thereby regulate multiple aspects of innate and adaptive immunity^2–5^. During the proliferative phase, E_2_ is present at low concentrations. However, E_2_ levels surge at ovulation, decline rapidly, before increasing again during secretory phase, when fertilization and implantation occur. In contrast, P levels remain low throughout the proliferative and ovulatory phases, before rising and peaking in mid-secretory phase. E_2_ and P act directly on FRT immune cells, epithelial cells and fibroblasts to regulate their function, and modulate the production of cytokines, chemokines and growth factors, which in turn regulate the innate and adaptive immune systems. These direct and indirect effects of sex hormones are essential for successful reproduction, and influence susceptibility to STIs in ways that are site-specific in the upper (endometrium, endocervix) and lower (ectocervix, vagina) FRT.

T cells are central to adaptive immune protection in the FRT and represent a major constituent of leukocytes (30–60%) in the endometrium^6–9^. To measure the cytotoxic potential of CD8+T cells, White et al. evaluated the ability of FRT mixed cell suspensions to kill targets using a redirected lysis assay^10^. In addition to demonstrating cytotoxic activity throughout the FRT, these studies demonstrated that endometrial CD3+CD8+T cell cytotoxic activity is markedly influenced by the stage of the menstrual cycle. Cytotoxic activity was elevated during the proliferative phase of the cycle and suppressed during the secretory phase. Interestingly, cytotoxic activity in the endometrium from postmenopausal women was significantly higher than activity at any point of the cycle in premenopausal women. Cycle dependent suppression occurred without changes in total CD8+T cell numbers. More recently, using a time-lapse imaging system to measure direct killing by purified CD8+T cells from the endometrium, we confirmed changes in cytotoxic activity during the menstrual cycle and increased activity following menopause^11^. Maximal suppression of CD8+T cell cytotoxic activity in the endometrium coincided with the time when fertilization and implantation is likely to occur, and therefore may represent a mechanism to avoid recognition and rejection of sperm and fetal cells by maternal endometrial CD8+T cells^1^. Unresolved however, is the extent to which hormonal changes during the menstrual cycle, act directly or indirectly to regulate CD8+T cells cytotoxic activity in the endometrium.

It is well known that sex hormones, which act both locally and systemically, have a spectrum of immunological effects on adaptive immunity and CD8+T cells in particular. E_2_ is known to affect the maturation, differentiation, and function of T cells. Others have shown that CD8+T cells express ER*α* and ER*β* receptors. For example, in rodents, E_2_ signaling through ER*α* was shown to be necessary for full differentiation of CD8+T cells^12,13^. In the human, retrovirally transduced peripheral blood mononuclear cells (PBMC) containing CD8+T cells incubated with E_2_ reportedly increased their expression of granzyme B, and IFN-γ^14^. In contrast, using human CD8+T cells, P has been shown to reduce IFN-γ production upon stimulation ex vivo, although the underlying mechanisms remain to be determined^15^. Beyond reduced production, other studies suggest that P impairs immune protective functions of antigen-non-specific CD8 T memory cells by inducing IFN-γ gene hyper-methylation^16^.

Beyond the direct effects of sex hormones, other soluble mediators are capable of modulating CD8+T cell cytotoxicity. For example, TGFβ inhibits intestinal CD8+T cell cytotoxicity through the suppression of perforin, without changes in T-bet, Eomes or granzyme B^17^. We recently reported that endometrial CD8+T cell cytotoxic function is also susceptible to suppression by TGFβ in both pre- and post-menopausal women, but that the inhibitory effect of TGFβ is reduced after menopause^11^. In animal studies, we found that TGFβ is produced by epithelial cells and fibroblasts from the FRT and under hormonal control in that E_2_ treatment increases the basolateral secretion of biologically active TGF*β* in the rodent^18,19^. However, the mechanisms by which TGFβ regulates CD8+T cell function in the endometrium remains to be elucidated.

The effects of sex hormones and TGF*β* on cytotoxic activity by CD8+T cells in the human have important implications for women’s health, especially as related to successful pregnancy, immunological infertility and protection against sexually transmitted diseases. The objective of this study was to evaluate the roles of E_2_, P and TGF*β* in regulating endometrial CD8+T cell cytotoxic capacity. Our results demonstrate that E_2_ and TGF*β* act directly on endometrial (EM) CD8+T cells to significantly suppress cytotoxic activity and intracellular perforin and granzyme A levels. In contrast, P has no direct effect on CD8+T cells but stimulates TGF*β* secretion by EM epithelial cells. These studies provide insight into mechanisms that control cytotoxic activity in the endometrium to increase reproductive potential.

## Results

### Estradiol and TGFβ suppress cytotoxic activity in CD8+T cells from the endometrium

**Figure 1:**
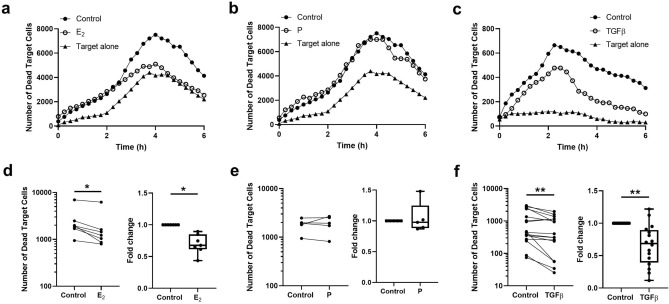
Effect of E_2_, P and TGFβ on endometrial CD8+T cells cytotoxicity. Purified endometrial CD8+T cells (effector cells, E) were pre-treated with E_2_ (5 × 10^−8^ M), P (1 × 10^−7^ M) for 48 h or TGFβ (10 ng/ml) for 2 h before co-culture with CFSE-stained allogeneic blood CD4+T cells (target cells, T) using a E:T ratio of 1:1. Cytotoxicity was measured over time by using quantitative time-lapse microscopy. Representative time-course of the kinetics of cytotoxicity over a period of 6 h in the absence (control) or presence of E_2_ (**a**), P (**b**) and TGFβ (**c**). Target alone are allogeneic blood CD4+T cells. Comparison of E_2_ (**d**, n = 7), P (**e**, n = 5) and TGFβ (**f**, n = 16) treatment in the mean number of dead target cells (left graph) and fold change in the mean number of dead target cells after E_2_, P and TGFβ treatment compared to control (right graph). Each dot represents a different patient. Min to Max (**d**–**f**, right graph) are shown. *P < 0.05; ** *P* < 0.01; Wilcoxon matched-pairs signed rank test.

We have previously demonstrated that cytotoxic activity of EM CD8+T cells is suppressed during the secretory phase of the menstrual cycle and enhanced after menopause^10,11^. In the present study, we investigated whether sex hormones are directly involved in the suppression of CD8+T cell cytotoxic activity. T cells were pretreated for 48 h with E_2_ or P at concentrations known to have physiological effects in vivo^20^ or with TGFβ for 2 h prior to measuring cell killing^11^. To measure cytotoxic activity, control and treated CD8+T cells were co-cultured with CFSE-labelled allogeneic target cells, and the number of dead cells quantified by time-lapse imaging as described before^11^. Based on our earlier studies for optimal killing, we selected an Effector:Target ratio of 1:1 and measured average cell killing during the first 6 h in culture. As seen in Fig. 1a–c, in representative time-course studies, relative to target cells alone, we detected a significant increase in dead target cells in the presence of allogeneic EM CD8+T cells. Moreover, when CD8+T cells were pretreated with E_2_ (Fig. 1a) or TGFβ (Fig. 1c), but not P (Fig. 1b), cell killing was markedly reduced. Figure 1d–f indicates the effect of hormones and TGFβ treatment on cytotoxicity measured both as total number of dead cells and as fold change in dead target cell numbers, the latter being done to compare experiments with different background mortalities of target cells alone. Consistent with our representative findings, relative to controls, E_2_ (Fig. 1d), and TGFβ (Figs. 1f), but not P (Fig. 1e), significantly reduced cell killing. Figure 1f confirms our earlier results^11^ that CD8+T cell cytotoxic activity is significantly reduced following TGFβ treatment of EM CD8+T cells.

### EM CD8+T cell expression of perforin and granzyme A is inhibited by E_2_

**Figure 2:**
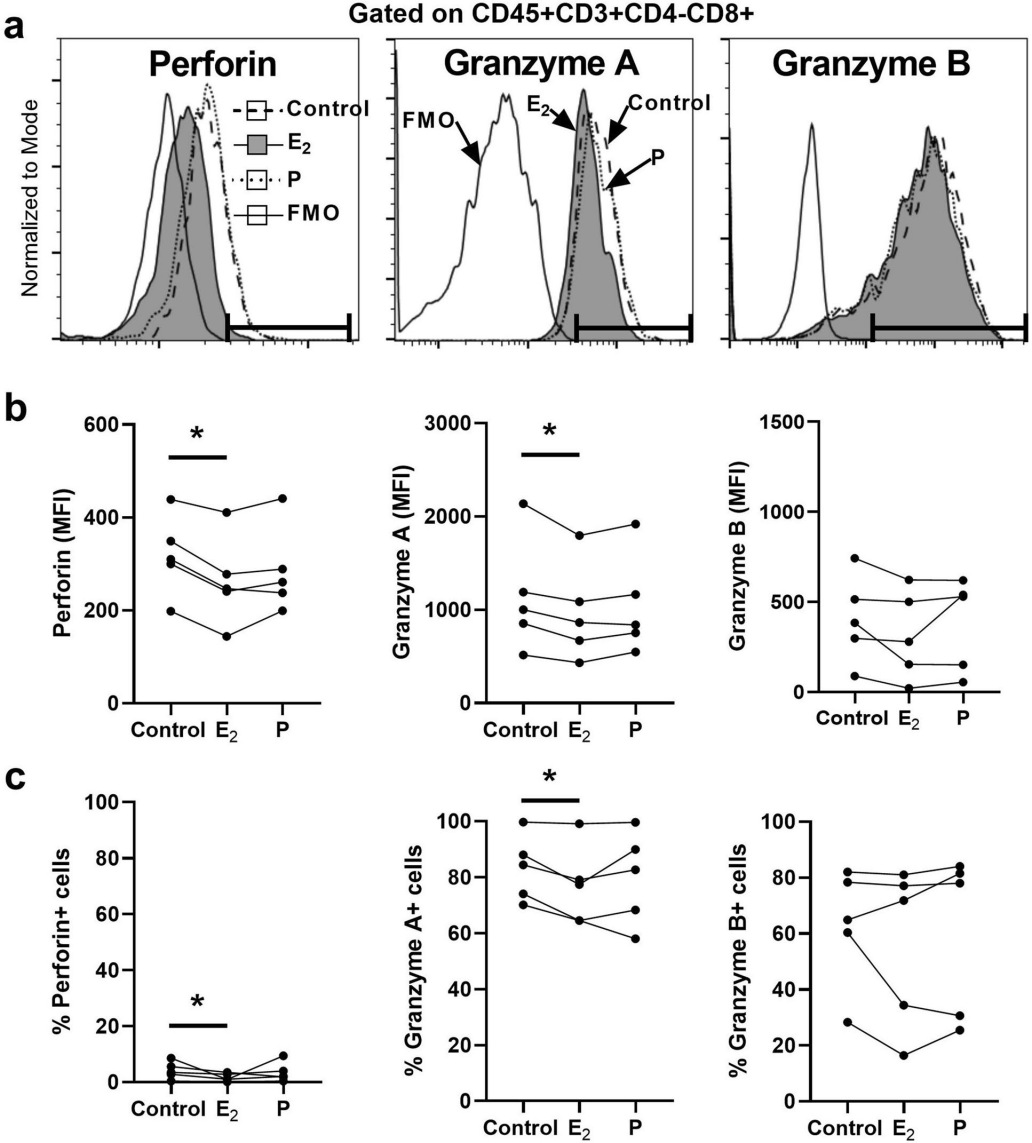
Effect of E_2_ and P on intracellular cytotoxic molecules in endometrial CD8+T cells. Mixed cell suspensions from endometrial tissues were pre-treated with E_2_ (5 × 10^−8^ M), P (1 × 10^−7^ M) for 48 h and stained for the intracellular cytotoxic molecules perforin, granzyme A and granzyme B for analysis by flow cytometry. (**a**) Representative histograms of intracellular perforin (left), granzyme A (middle) and granzyme B (right) expression in endometrial CD8+T cells treated with E_2_ and P. Gate boundaries (horizontal line) were set by fluorescence minus one (FMO) to indicate the gating of positive cells. (**b**) Median fluorescence intensity (MFI) of perforin (left), granzyme A (middle), granzyme B (right) and (**c**) percentage of perforin+ (left), granzyme A+ (middle), granzyme B+ (right) in endometrial CD8+T cells following incubation with E_2_ and P. Each dot represents a different patient (n = 5). *P < 0.05; Friedman test with Dunn’s multiple comparisons test.

To identify the underlying mechanisms involved in suppression of cytotoxic T cell activity, we evaluated the expression of perforin, granzyme A (GZA) and granzyme B (GZB) following preincubation of CD8+T cells with E_2_ or P. EM tissues were digested to obtain mixed cell suspensions and stained for intracellular cytotoxic molecules prior to analysis by flow cytometry as previously described^11^. Figure 2a contains representative histogram overlays of CD8+T cells for perforin, GZA and GZB indicating the downward shift in expression of perforin and GZA in response to E_2_ but not P. Analysis of five separate experiments with different subjects (Fig. 2b), demonstrated that E_2_ significantly reduced the intracellular content (MFI) of perforin and GZA in CD8+T cells. In contrast, E_2_ had no significant effect on GZB. Hormone treatment also affected the percentage of cells expressing cytotoxic molecules, as seen in Fig. 2c, with a significant decrease in the percentage of cells positive for perforin and GZA cells following incubation with E_2_. Overall, these studies demonstrate that E_2_ decreases the expression of perforin and GZA in EM CD8+T cells, which correlates with reduced cell cytotoxicity. Further, it indicates that these changes are hormone specific given that P had no effect on either perforin, GZA, and GZB intracellular expression or the percentage of positive cells.

### EM CD103+and CD103- CD8+T cell expression of perforin and granzyme A is inhibited by estradiol

**Figure 3:**
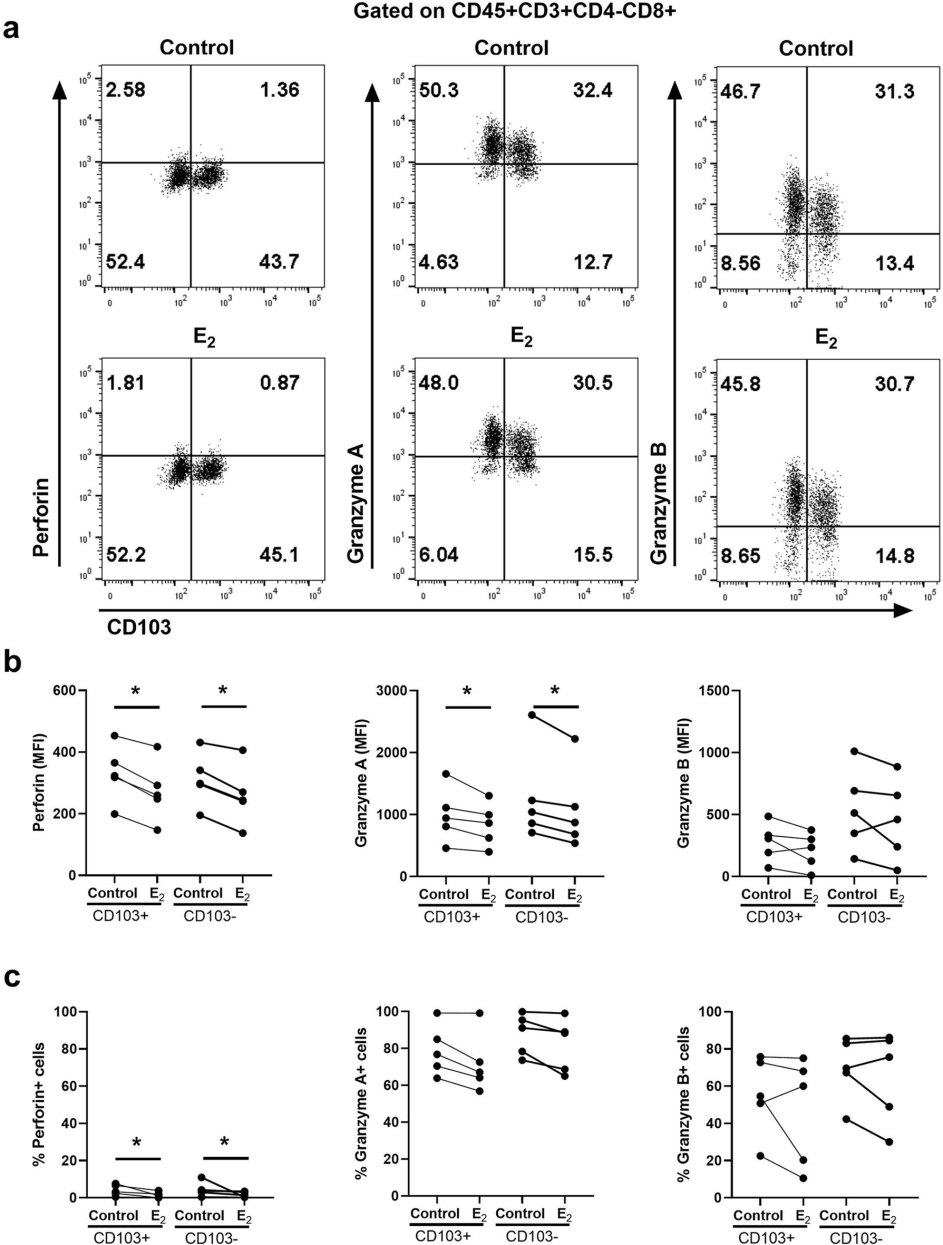
Effect of E_2_ on intracellular cytotoxic molecules in endometrial CD103+ and CD103−CD8+T cells. Mixed cell suspensions from endometrium were pre-treated with E_2_ (5 × 10^–8^ M) for 48 h and stained for the intracellular cytotoxic molecules perforin, granzyme A and granzyme B for analysis by flow cytometry. (**a**) Representative dot plots of intracellular perforin (left), granzyme A (middle) and granzyme B (right) expression in endometrial CD103+and CD103−CD8+T cells treated with E_2_. Top row shows control group (without E_2_) and bottom row shows E_2_ treated group. (**b**) Median fluorescence intensity (MFI) of perforin (left), granzyme A (middle), granzyme B (right) and (**c**) percentage of perforin+ (left), granzyme A+ (middle), granzyme B+ (right) in endometrial CD103+and CD103−CD8+T cells following incubation with E_2_. Each dot represents a different patient (n = 5). *P < 0.05; Friedman test with Dunn’s multiple comparisons test.

In previous studies, we demonstrated that human EM CD8+T cells consist of tissue resident cells and non-resident CD8+T cells that are CD103+and CD103−, respectively^8,11^. When cytotoxicity was compared between CD103+and CD103−CD8+T cells, direct cytotoxic activity and expression of cytotoxic molecules was lower in CD103+compared to CD103−T cells^11^. Therefore, in this study we explored whether the observed effects of E_2_ in reducing cytotoxic molecule expression were due to effects on both CD103+and CD103−T cell populations. Mixed cell suspensions from EM tissues were incubated with E_2_ for 48 h prior to measuring granular content by flow cytometry. Figure 3a shows a representative example of the content of perforin, GZA and GZB before and after treatment with estradiol. As shown in Fig. 3b, using matched samples from multiple patients, E_2_ significantly suppressed the content of perforin and GZA (MFI) in both tissue resident (CD103+) and non-resident (CD103−) CD8+T cells. In contrast, E_2_ had no effect on GZB. Interestingly, as seen in Fig. 3c, E_2_ reduced the percent positive CD103+and CD103−CD8+T cells containing perforin but had no effect on the percentage of cells that were GZA or GZB positive. These findings suggest that E_2_ regulates both CD103+and CD103−CD8+T cells and that suppression of the levels of cytotoxic molecules is unique in that each of the cytotoxic responses to E_2_ are separate and distinct. This is consistent with our previous results that menopausal status equally affects resident and non-resident T cells^11^, suggesting that tissue residency does not modify sensitivity to hormonal control.

### Perforin, granzyme A and B Content in EM CD8+T Cells is suppressed by TGFβ

**Figure 4:**
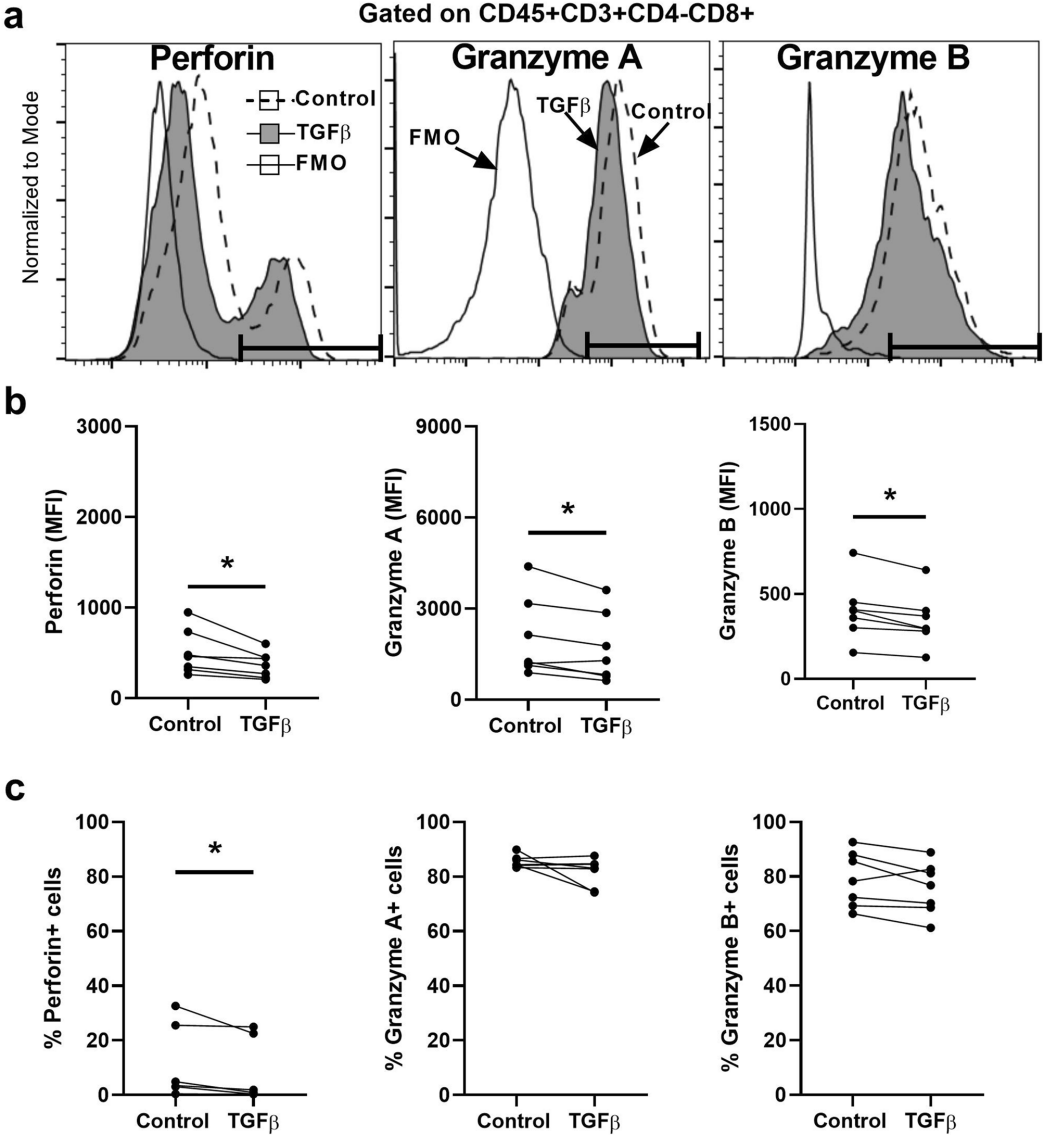
Effect of TGFβ on intracellular cytotoxic molecules in endometrial CD8+T cells. Mixed cell suspensions from endometrial tissues were pre-treated with TGFβ (10 ng/ml) for 48 h and stained for the intracellular cytotoxic molecules perforin, granzyme A and granzyme B for analysis by flow cytometry. (**a**) Representative histograms of intracellular perforin (left), granzyme A (middle) and granzyme B (right) expression in endometrial CD8+T cells treated with TGFβ. Gate boundaries (horizontal line) were set by fluorescence minus one (FMO) to indicate the gating of positive cells. (**b**) Median fluorescence intensity (MFI) of perforin (left), granzyme A (middle), granzyme B (right) and (**c**) percentage of perforin+ (left), granzyme A+ (middle), granzyme B+ (right) in endometrial CD8+T cells following incubation with TGFβ. Each dot represents a different patient (n = 7). *P < 0.05, Wilcoxon matched-pairs signed rank test.

**Figure 5:**
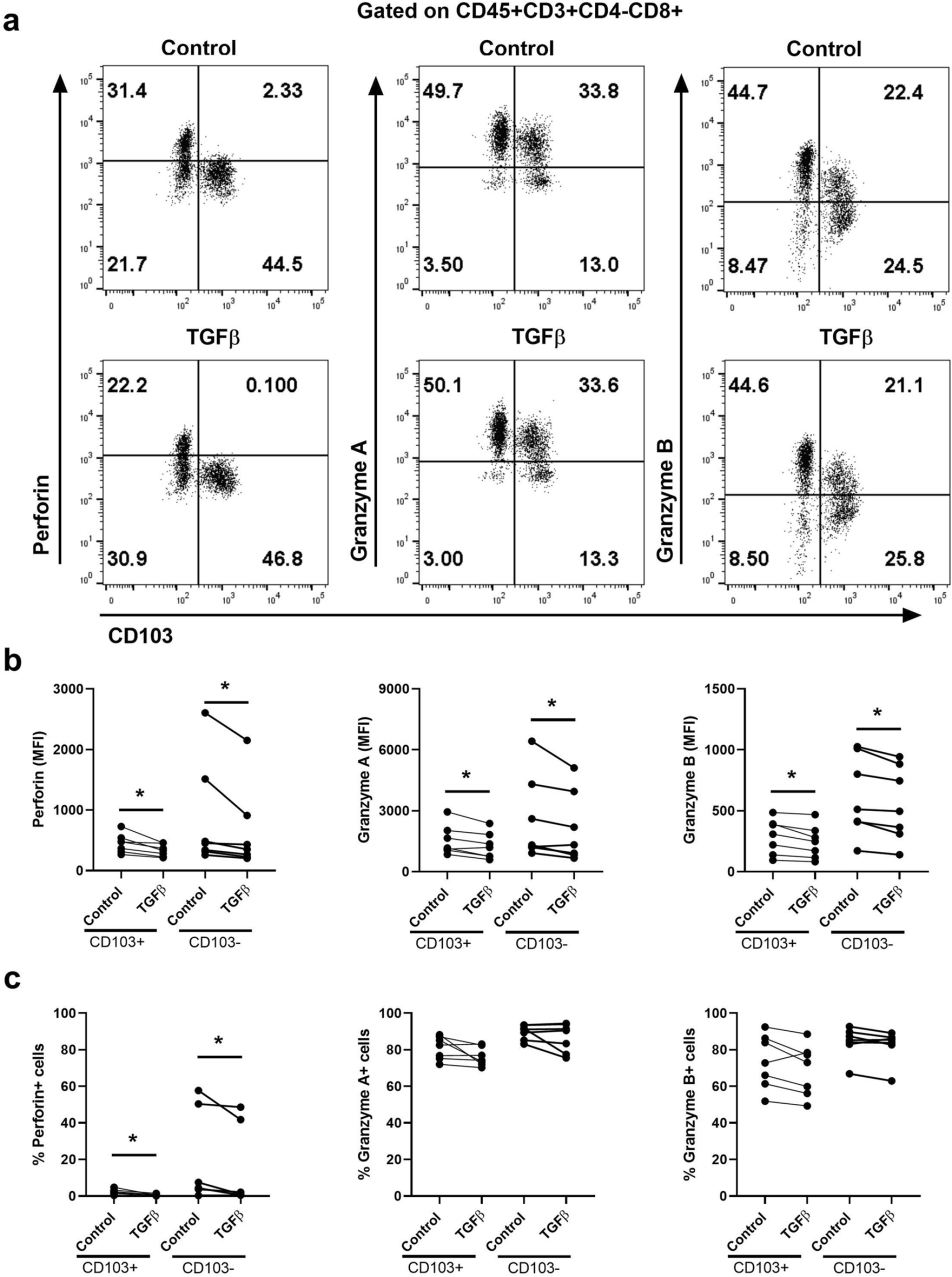
Effect of TGFβ on intracellular cytotoxic molecules in endometrial CD103+ and CD103-CD8+T cells. Mixed cell suspensions from endometrial were pre-treated with TGFβ (10 ng/ml) for 48 h and stained for the intracellular cytotoxic molecules perforin, granzyme A and granzyme B for analysis by flow cytometry. (**a**) Representative dot plots of intracellular perforin (left), granzyme A (middle) and granzyme B (right) expression in endometrial CD103+and CD103−CD8+T cells treated with TGFβ. Top row shows control group (without TGFβ) and bottom row shows TGFβ treated group. (**b**) Median fluorescence intensity (MFI) of perforin (left), granzyme A (middle), granzyme B (right) and (**c**) percentage of perforin+ (left), granzyme A+ (middle), granzyme B+ (right) in endometrium CD103+ and CD103-CD8+T cells following incubation with TGFβ. Each dot represents a different patient (n = 7). *P < 0.05; Friedman test with Dunn’s multiple comparisons test.

Next, we investigated the underlying mechanisms of TGFβ-mediated cytotoxicity suppression. CD8+T cells in mixed cell suspensions were incubated with TGFβ prior to perforin, GZA, and GZB analysis by flow cytometry at 48 h. As seen in the histograms (Fig. 4a) and analyses of intracellular content (Fig. 4b), TGFβ significantly reduced the concentration of all three intracellular cytotoxic molecules. In our patients we observe a wide range of perforin expression from 0.5 to 32.6% as shown in this study and in our previous publication^11^. The representative plots presented in Figs. 2 and 4 are from two different patients. Figure 2 is from a patient with low perforin expression, while Fig. 4 is from a patient with high perforin expression. These profiles are examples that reflect the range in expression profiles between patients. We also found that TGFβ reduced the percentage of total CD8+T cells that were perforin positive, but had no effect on the percentage of cells expressing GZA and GZB (Fig. 4c). We next investigated the effect of TGFβ on cytotoxic molecules in CD103+and CD103−CD8+T cell compartments (Fig. 5a). As seen in Fig. 5b, TGFβ significantly reduced the expression of perforin, GZA and GZB within both CD103+and CD103−CD8+T cell populations. Interestingly, TGFβ reduced the number of perforin positive cells but had no effect on cells positive for GZA or GZB (Fig. 5c).

### Progesterone increases the basolateral secretion of TGFβ by polarized EM epithelial cells

**Figure 6:**
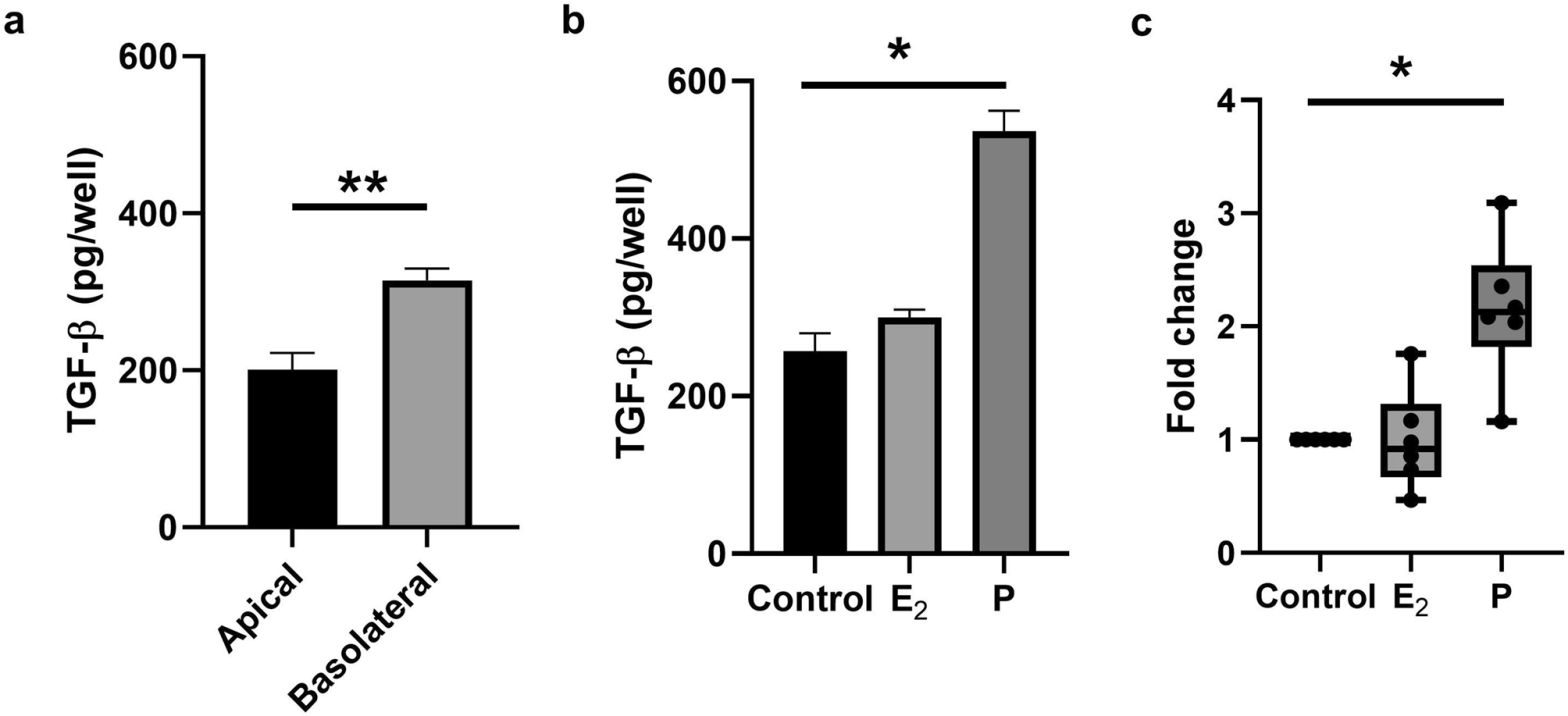
Constitutive production and regulation of TGFβ secretion in endometrial epithelial cells. Endometrial epithelial cells were isolated and cultured until confluence and high transepithelial resistance was reached. Polarized endometrial epithelial cells were treated with E_2_ (5 × 10^−8^ M) or P (1 × 10^−7^ M) for 48 h. Apical and basolateral conditioned media (CM) were collected and assayed total TGFβ by ELISA. (**a**) Level of total TGFβ secreted following 48 h accumulation of constitutive apical and basolateral (n = 14). (**b**) Representative experiment showing the basolateral secretion of TGFβ by polarized endometrial epithelial cells following treatment with E_2_ or P for 48 h. (**c**) Graph represents the fold change in total TGFβ in basolateral CM following E_2_ or P treatment compared with untreated controls (n = 6). The mean and SEM (**a** and **b**) and Min to Max (**c**) are shown. *P < 0.05; ** *P* < 0.01; Wilcoxon matched-pairs signed rank test (**a**) and Friedman test with Dunn’s multiple comparisons test (**b** and **c**).

In addition to their directs effects, sex hormones are known to indirectly regulate immune cells by acting on adjacent cells in the tissue environment. TGFβ is known to be synthesized and secreted by EM epithelial cells and stromal cells from the uterus^18,19^. Our studies in a rodent model demonstrated that polarized EM epithelial cells preferentially secrete biologically active TGFβ into the basolateral compartment^18^. Therefore, we analyzed TGFβ production by polarized human EM epithelial cells. As seen in Fig. 6a, TGFβ was secreted constitutively by polarized epithelial cells with preferential release basolaterally, in the direction of underlying tissues. Since we have previously demonstrated that human polarized epithelial cells are hormonally responsive to E_2_^21^, we hypothesized that E_2_ might increase TGFβ secretion by epithelial cells as a secondary mechanism for regulating CD8+T cell cytotoxic activity. To test this, polarized EM epithelial cells were grown to confluence and incubated with E_2_ or P for 48 h prior to collection of basolateral media. Unexpectedly, as shown in a representative experiment (Fig. 6b), we found that P increased the basolateral secretion of TGFβ relative to controls and that E_2_ had no effect on TGFβ production. To more fully analyze this response, we normalized the data from 6 experiments to express TGFβ production as the fold change relative to control cells, to compare experiments with different secretion backgrounds by control cells. As shown in (Fig. 6c), following normalization, P but not E_2_ consistently stimulated the secretion of TGFβ by EM epithelial cells. Overall, these studies suggest that during the secretory phase menstrual cycle, under the influence of progesterone, EM epithelial cells secrete increased amounts of TGFβ basolaterally into the tissue compartment to suppress CD8+T cell cytolytic activity.

## Discussion

**Figure 7:**
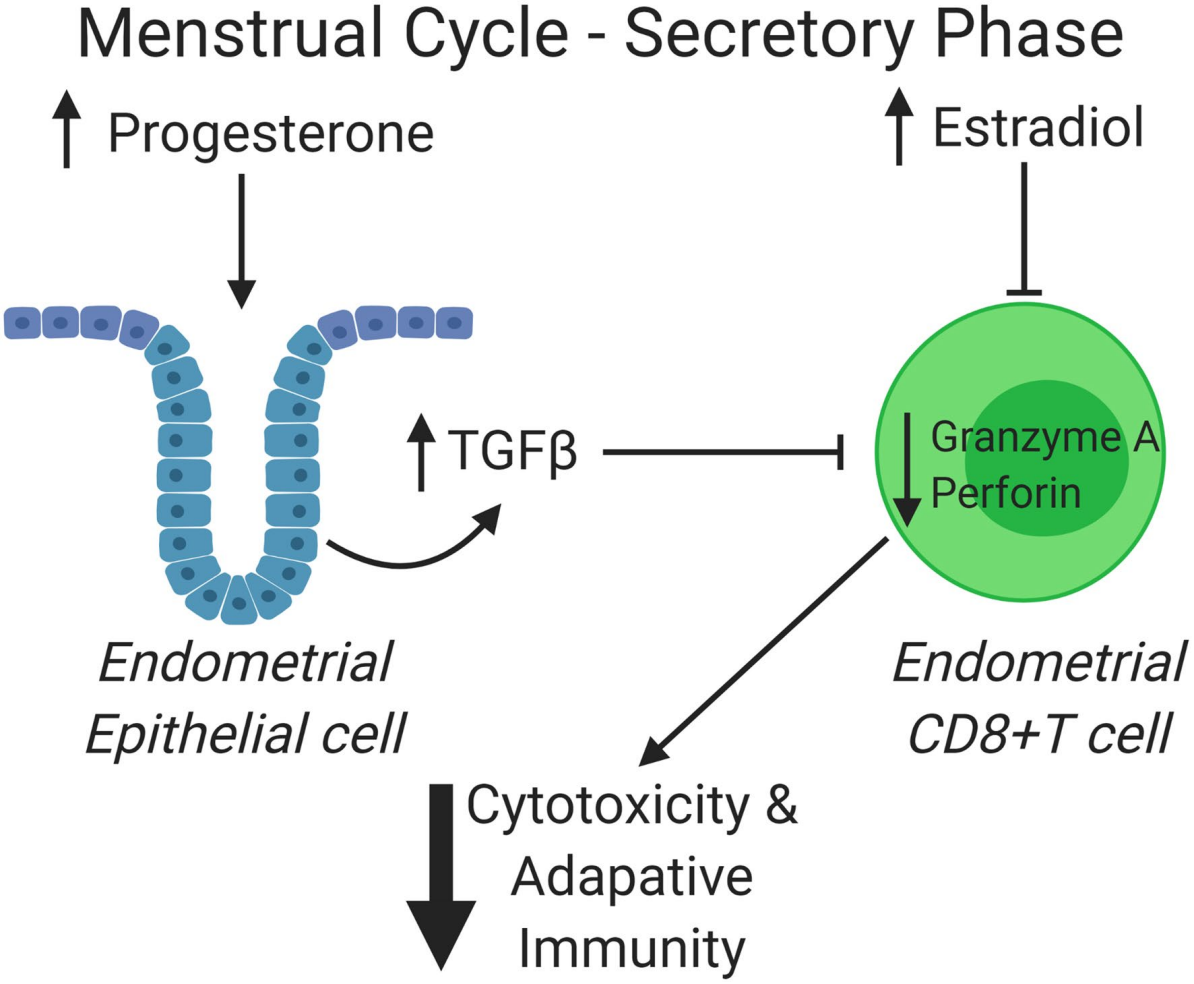
Estradiol and progesterone regulate CD8+T Cell cytotoxic activity in the endometrium during the secretory phase of the menstrual cycle. Schematic illustrating the complex relationship between sex hormones and CD8+T cells in the uterine endometrium during the secretory phase of the menstrual cycle^1^. Based on findings in this paper, E_2_ and *P* play a central role in down-regulating adaptive immunity which optimizes the conditions for successful pregnancy^22^. Progesterone, for example, is synthesized by the ovary and peaks following ovulation in preparation for implantation. Our findings indicate that in response to *P*, but not E_2_, epithelial cells secrete TGFβ into the basolateral (tissue) compartment adjacent to CD8+T cells thereby reducing the intracellular content of perforin and GZA and cytolytic potential. Interestingly, *P* had no direct effect of CD8+T cells. Accompanying the indirect effect of *P*, E_2_, which is known to peak at ovulation, decline and then rise throughout the secretory phase of the cycle^23^, through its direct effect on CD8+T cells, decreases perforin and GZA content thereby suppressing endometrial CD8+T cell adaptive activity.

Our study demonstrates that E_2_ and TGFβ directly suppress EM CD8+T cell cytotoxic activity by downregulating intracellular perforin, GZA, or GZB expression. Further, we demonstrate that whereas E_2_ acts directly on CD8+T cells to suppress killing, P has no direct effect. However, P upregulates TGFβ secretion by EM epithelial cells, which could represent an indirect mechanism of control of cytotoxic activity in vivo. Overall, as depicted in Fig. 7, our findings demonstrate direct and indirect hormone-dependent mechanisms in the human endometrium able to suppress CD8+T cell cytolytic capacity in premenopausal women during the secretory phase of the menstrual cycle, without which successful implantation and pregnancy would be unlikely to occur.

CD8+T cells are key effector cells for immune protection, but their function in the endometrium is tightly regulated to avoid rejection of the semi-allogeneic fetus^1,24^. Our previous studies demonstrate that EM CD8+T cell cytotoxic activity is suppressed in premenopausal women compared to postmenopausal women, particularly during the secretory phase of the menstrual cycle, suggesting that sex hormones are key regulators of CD8+T cell cytotoxicity^10,11^. However, the direct effect of sex hormones in suppression of cytotoxic function of EM CD8+T cells had never been addressed before. Therefore, we investigated whether sex hormones directly contribute to the suppression of CD8+T cell cytotoxic activity seen in premenopausal women.

We found that E_2_ directly suppresses cytotoxic activity of EM CD8+T cells, and that the reduction in killing capacity is due to decreased expression of the cytotoxic molecules perforin and GZA in CD8+T cells. In contrast to our studies which showed no effect of E_2_ on GZB expression, Navarro et al.^14^ found that E_2_ increased the expression of GZB in blood. Differences observed are most likely due to their use of buffy-coat blood suspension containing CD8+T cells that were retrovirally transduced and activated, whereas we used purified EM CD8+T cells without any in vitro activation stimulus to measure the effects of E_2_. Furthermore, to test GZB production, they used a preparation of total T cells, containing both CD4+and CD8+T cells, while we used purified CD8+T cells. Whether activation status alters EM CD8+T cell responsiveness to E_2_ remains to be determined.

In contrast to E_2_, we found that P had no direct effect on EM CD8+T cell cytotoxic activity or cytotoxic granule content. This lack of effect is probably due to the previously reported absence of nuclear P receptors in CD8+T cells^25^ and contrasts with findings showing P changes in cytokine production and reduction in T-cell proliferation following P treatment of peripheral blood CD8+T cells, which the authors attributed to P membrane receptors^15^. These differences suggest a finite specificity of P effects that are unique to the class of P receptor present or to functional differences between peripheral blood CD8+T cells and EM CD8+T cells.

Confirming our previous results, we found that EM cytotoxic function was rapidly suppressed by TGFβ after short-time treatment^11^. In the present study, we demonstrate that the suppressive effects of TFGβ are due to a reduction in the content of intracellular cytotoxic molecules (perforin, GZA and GZB). Given that in our experimental setting, cells were pre-treated with TGFβ for 2 h prior to cytotoxicity analysis and 48 h prior to intracellular cytotoxic molecule assessment, the underlying mechanism for rapid regulation of cytotoxic capacity remains unclear, and further investigation is warranted. Others have demonstrated that perforin and granzymes work synergistically to induce apoptosis in target cells recognized by CD8+T cells^26^. In addition to reductions in GZA and GZB, our findings show a reduction in both the levels of intracellular content of perforin as well as the percent positive cells. This is consistent with reports by others showing that cytotoxic function is suppressed by TGFβ through the reduction of perforin positive CD8+T cell numbers in the gastrointestinal tract^17^, and may indicate a general mechanism by which TGFβ controls cytotoxic activity of CD8+T cells in tissues. To the best of our knowledge, our findings are the first to demonstrate that E_2_ and TGFβ in culture reduce both the number of percent perforin positive CD8+T cells as well as the intracellular content of cytotoxic molecules of these cells.

We and others have demonstrated that TGFβ is produced by epithelial cells and fibroblasts from the FRT and under hormonal control in a rodent model^18,19^. Interestingly, in the human, TGFβ mRNA expression in the endometrium is increased during the secretory phase of the menstrual cycle^27^, when EM CD8+T cells cytotoxic activity is maximally suppressed^10,11^. Our current study extends these findings by demonstrating that P acts directly on human EM epithelial cells to stimulate the secretion of TGFβ. Interestingly, we found that E_2_ had no effect on epithelial cell TGFβ production, in contrast to our previous findings with the rodent model^18^. Consistent with our results, early studies in non-human primates also found that P increased mRNA of TGFβ2 in the endometrium^28^. We have previously demonstrated broad effects of E_2_ treatment on epithelial cells, including stimulation of intracellular genes, such as Cytosolic 5′-nucleotidase and P receptor^29^, and the secretion of SLPI along with other antimicrobial peptides with broad antimicrobial, antiviral and antifungal activity^21,30^. Our findings in the present study highlight the selectivity of hormonal effects on epithelial cells. Whether after its secretion in response to P, other hormonally dependent steps further process TGFβ to increase biological activity remains to be determined^31,32^. Taken together, these findings suggest E_2_ and P precisely regulate adaptive immunity in the endometrium, by acting indirectly on epithelial cells to increase the production of TGFβ.

The menstrual cycle is divided into a proliferative and a secretory phase, during which fertilization and implantation occurs. Others have shown that shortly after ovulation, during the secretory phase of the menstrual cycle, levels of P rise sharply and are sustained for 10–12 days^33^. Accompanying this increase in P is a secondary mid-secretory rise in E_2_ which is essential for implantation. In contrast, the proliferative phase of the cycle is dominated by E_2_. These hormonal changes control the cyclical remodeling of the endometrium in premenopausal women to optimize conditions for implantation. Based on our findings in the present study, we propose that in premenopausal women, E_2_ directly suppresses cytotoxic activity by EM CD8+T cells and reduces the intracellular content of perforin and GZA. With ovulation and unique to the endometrium, P induces the production of TGFβ by epithelial cells, which in turn acts on EM CD8+T cells to further suppress cytotoxic activity, and reduce the expression of perforin, GZA and GZB, achieving maximum suppression during the secretory phase of the menstrual cycle (Fig. 7).

In our previous studies, we demonstrated that cytotoxic activity is significantly increased in postmenopausal women^10,11^. Following menopause, sex hormones levels (E_2_ and P) drop and remain low and constant. Our results in this study suggest that the reduction in sex hormones removes the direct and indirect suppressive mechanisms from EM CD8+T cells, resulting in an increase in cytotoxic activity and intracellular content of cytotoxic molecules, consistent with our previous findings^11^.

We and others have shown that CD8+T cells in the endometrium consist of both CD103+and CD103−T cells, likely representing tissue resident and non-resident memory T cells respectively. We demonstrated that cytotoxic activity and intracellular content of cytotoxic molecules was significantly higher in CD103−compared to CD103+EM CD8+T cells^11^. Here we investigated whether the regulatory effects of E_2_ and TGFβ were preferential to either subset. We found that E_2_ and TGFβ affected CD103+and CD103−CD8+T cells equally and reduced the content of cytotoxic molecules in both subsets. These findings suggest that tissue-resident and non-resident CD8+T cells are equally susceptible to regulation by sex hormones, and that transition into tissue-residency status does not modify sensitivity to regulation by sex hormones and the tissue environment. Our results here are also in agreement with our previous observation that postmenopausal women tended to have decreased expression of GZB and increased expression of GZA compared to premenopausal women in both CD103−and CD103+CD8+T cells^11^. This would suggest that removal of sex hormone suppression after menopause induces changes in intracellular content of cytotoxic molecules in both cellular subsets.

Given that cytotoxic activity following menses returns during the follicular phase of the menstrual cycle^10,11^, suggests either that the absence of P and TGFβ reverses intracellular granular content and cytolytic activity or that a new population of CD8+T cells enters the endometrium. That cytolytic potential can be restored was suggested from studies in which in vitro neutralization of TGFβ partially restored perforin expression in gut CD8+T cells^17^. Moreover, using an inhibitor that blocks TGFβ signaling in EM CD8+T cells, we rescued cytotoxic activity in some samples^11^. Whether cytolytic potential is restored as hormone levels change with the menstrual cycle or the result of recruitment of CD8+T cells to the endometrium with the start of a new menstrual cycle remains to be determined. Further elucidation of EM cytotoxic T lymphocyte (CTL) activity and its implications for successful reproduction and protection against sexually transmitted diseases, including HIV, will provide essential information for the development of vaccines and immunotherapies targeted to the FRT.

Regarding study limitations, in addition to the mechanisms addressed in our study, alternative mechanisms of regulation of cytotoxic activity by sex hormones remain to be explored. For example, while we detected significantly lower levels of cytotoxic molecules following E_2_ treatment, whether the observed effects were due to direct actions of E_2_ on the production of cytotoxic molecules or secondary to decreased activation status of CD8+T cells remains to be determined. Further, in addition to the perforin/granzyme pathway, the FAS/FASL pathway also mediates cell death^34^. In preliminary studies we have observed no modification of sFASL production by EM CD8+T cells after incubation with hormones under resting conditions. Further research is needed to address whether the FAS/FASL pathway is significantly modified by E_2_ after T cell stimulation, and if so, the potential contribution of the FAS/FASL pathway to cytotoxic function of EM CD8+T cells.

Another important aspect not addressed in this study is the extent to which sex hormones modify degranulation of T cells. In previous studies, we demonstrated a significant increase in degranulation capacity of CD8+T cells from the FRT after menopause^11^, suggesting a potential implication of sex hormones in regulation of degranulation capacity. Future studies are needed to investigate the extent to which E_2_ and P directly modify degranulation of CD8+T cells. Furthermore, in addition to cytotoxic molecules, FRT CD8+T cells can secrete and array of cytokines and chemokines upon stimulation, including IFN-γ and TNFα^35^. In preliminary studies, we observed no differences in secretion of IFN-γ and TNFα in CD8+T cells treated with E_2_ or P under resting conditions; however, whether cytokine production after stimulation is modified by sex hormones remains to be addressed in future studies.

Although our study is focused on CD8+T cells, a full understanding of the regulation of cytotoxic activity in the FRT during the menstrual cycle will require the functional evaluation of CD4+T cells and NK cells. We have previously demonstrated decreased proportions of CD4+T cells in the EM from postmenopausal women^9^. However, whether E_2_ and P modify the presence and function of EM CD4+T cells throughout the menstrual cycle is unknown. Endometrial NK cells, which have overlapping cytotoxic functions with CD8+T cells, increase in numbers from approximately 3% of leukocytes in proliferative phase to 30% in secretory phase tissues^36–38^ where they surround the spiral arteries^39^, due to increased chemotaxis and proliferation. Whether sex hormones directly influence cytotoxic function of endometrial NK cells is unclear. Previous studies using peripheral blood NK cells have shown contradictory evidence of a menstrual cycle effect on cytotoxicity^40,41^. However, others have demonstrated that endometrial NK cells in the early proliferative phase have lower cytotoxicity compared to other stages of the menstrual cycle^42^. Together these findings suggest that sex hormones modulate cytotoxic activity of multiple cell types as part of a larger program to optimize conditions in the FRT for successful reproduction.

In conclusion, our study demonstrates two unique hormone-dependent regulatory mechanisms that regulate CD8+T cell cytotoxic function in the uterine endometrium, one which involves the direct effect of E_2_ and the other, the indirect effect of P-mediated epithelial cell production of TGFβ. In response to E_2_ and TGFβ, CD103+and CD103−CD8+T cell cytolytic potential is suppressed. These results are central to our understanding of hormonal regulation of cytotoxic activity during the menstrual cycle, during pregnancy and following menopause, and to the development of therapeutics for both the prevention and control sexually transmitted infections and gynecological cancers.

## Methods

### Study subjects

Studies were performed with Dartmouth College Institutional Review Board approval. All investigations were conducted according to the principles expressed in the Declaration of Helsinki and carried out with the approval to use discarded tissues from hysterectomies from the Committee for the Protection of Human Subjects (CPHS), Dartmouth-Hitchcock Medical Center, and with written informed consent obtained from the patients before surgery. Indications for surgery were benign conditions such as fibroids and prolapse (age from 31 to 82); tissue samples selected were distant from the sites of pathology and without pathological lesions as determined by a pathologist. Women were not on oral contraceptives before hysterectomy. Menopausal status was determined by a pathologist based on the histological evaluation of sections of the endometrium (endometrial dating). Post-menopausal status was characterized by an atrophic endometrium. Information regarding genital infections was not available. Blood Leuko Paks from women were obtained from our IRB-approved collection facility at Dartmouth–Hitchcock Medical Center. Blood donors were anonymous, no information regarding age or hormonal status was available and only female donors were used in this study.

### Tissue processing

EM tissues were transferred to the laboratory immediately after surgery and processed as previously described^8,9,43–45^. Average tissue weight obtained was 2.6 ± 2.0 g. Tissues were rinsed with 1 × HBSS (Hanks balanced salt solution) supplemented with phenol red, 100 U/ml penicillin, 100 µg/ml streptomycin (all Thermo Scientific Hyclone, Logan, UT), and 0.35 mg/ml NaCO_3_ (Fisher Scientific, Pittsburgh, PA). Tissues were then minced under sterile conditions into 1–2 mm fragments and digested using an enzyme mixture containing 0.05% collagenase type IV (Sigma-Aldrich, St. Louis, MO) and 0.01% DNAse (Worthington Biochemical, Lakewood, NJ) for 1 h at 37ºC. Type IV collagenase was selected based on preliminary studies to ensure non-cleavage of surface markers^9,44^. After digestion, cells were dispersed through a 250-µm nylon mesh screen (Small Parts, Miami Lakes, FL), washed, and resuspended in complete media consisting of DMEM/F12 medium without phenol red (Invitrogen Life Technologies, Carlsbad, CA) supplemented with 20 mM HEPES, 2 mM L-glutamine (Invitrogen Life Technologies, Carlsbad, CA), 50 mg/mL primocin (Invivogen, San Diego, CA, USA) and 10% heat-inactivated defined Fetal Bovine Serum (FBS) (Hyclone, Logan, UT, USA). Epithelial cell sheets were separated from stromal cells by filtration through a 20-µm mesh filter (Small Parts). Epithelial cell sheets were retained on the filter, while stromal cells passed through. Stromal cells were then washed, counted and dead cells removed using the Dead cell removal kit (Miltenyi Biotec, Auburn, CA) according to manufacturer instructions. The resulting mixed cell suspension, consisting of immune cells and stromal fibroblasts, was used for flow cytometric analysis and further CD8+T cells purification.

### Isolation and culture of EM epithelial cells

Epithelial cell sheets were recovered from 20-µm mesh filters by rinsing and backwashing the filter with complete medium, centrifuged at 500 × *g* for 5 min and analyzed for cell number and viability. To establish a cell culture system of polarized human EM epithelial cells with both apical and basolateral compartments, EM epithelial cells were cultured in Matrigel matrix (BD Biosciences) coated Falcon cell culture inserts in 24-well companion culture plates (Fisher Scientific). Apical and basolateral compartments contained 300 and 500 µl of complete medium, respectively, which was changed every 2 days. Tight junction formation of epithelial cell monolayers from EM was assessed by periodically measuring transepithelial resistance (TER) using an EVOM electrode and Voltohmmeter (World Precision Instruments, Sarasota, FL), as described previously^46–48^.

### Preparation of EM CD8+T cells

Following removal of dead cells from the filter pass through suspension of stromal cells, CD8+T cells were isolated using negative magnetic bead selection with the CD8+T cell isolation kit (Miltenyi Biotec) following instructions with minor modifications. This negative selection protocol delivers untouched CD3+CD8+T cells. Additionally, anti-fibroblast microbeads (Miltenyi Biotec) were added in combination with the microbeads supplied with the kit to ensure depletion of stromal fibroblasts present in the mixed cell suspension as described before^9,11^. After two rounds of negative selection, purity of the CD8+T cell population was higher than 90%, with approximately 2% contamination with non-immune cells, 2% CD3- cells and 1–2% contamination with CD4+T cells^11^. The average number of total CD8+T cells recovered per gram of tissue was 2 × 10^5^. Following isolation, purified CD8+T cells were resuspended in immune cell media consisting of X-VIVO 15 Media (Lonza, Walkersville, MD) supplemented with 10% charcoal stripped human AB serum (Valley Biomedical, Winchester, VA) and co-cultured with E_2_ and/or P for 48 h or TGFβ (10 ng/ml, PeproTech Inc.)^11,49^ for 2 h prior to cytotoxicity assays.

### Preparation of blood CD4+T cells

Blood CD4+T cells were purified using negative magnetic beads selection with the CD4+T cell isolation kit (Miltenyi Biotech) following isolation of PBMC by standard Ficoll density gradient centrifugation^9,50^. Freshly isolated blood CD4+T cells were stained with CFSE (Cell Division Tracker Kit; BioLegend) as recommended by the manufacturer. The cells were then resuspended in immune cell media as allogeneic target cells prior to cytotoxicity assays.

### Hormone preparation

17β-estradiol (E_2_, Calbiochem, Gibbstown, NJ) or progesterone (P, Calbiochem) was dissolved in 100% ethanol for an initial concentration of 1 × 10^−3^ M, evaporated to dryness and suspended in epithelial cells complete media containing 10% charcoal dextran-stripped FBS or immune cell complete media to a concentration of 1 × 10^−5^ M. Further dilutions were made to achieve final working concentration, and cells were treated with 5 × 10^−8^ M E_2_ and/or 1 × 10^−7^ M P. Both are standard hormone treatment concentrations used by our laboratory and each is within the physiological range of hormone concentration^20^. As a control, an equivalent amount of ethanol without dissolved hormone was initially evaporated.

### Sex hormone treatment and TGFβ analysis of EM epithelial cells

Epithelial cells in culture were switched to complete media containing 10% of charcoal dextran-stripped FBS prior to hormone treatment. After 24 h, the media was replaced, and cells were treated with hormone for 48 h. Hormone (E_2_ or P) or ethanol control media was added to both the apical and basolateral compartments. After 48 h incubation, apical and basolateral conditional media (CM) were collected and stored immediately at−80ºC prior to TGFβ ELISA (R&D systems, Minneapolis, MN) according to the manufacturer’s recommendations.

### Cytotoxicity assay

Purified and preincubation of CD8+T cells with E_2_, P or TGFβ were co-cultured with CFSE-stained blood CD4+T cells in immune cell media, at an Effector:Target ratio of 1:1, in 96 well plates. Cytotox red (IncuCyte Cytotox Red, Essen Bioscience) was added to the media to stain dead cells. Plates were imaged every 15 min using the IncuCyte Zoom system (Essen Bioscience), and dead target cells were automatically quantified over time as double green (CFSE) and red (Cytotox) stained cells^11^.

### Flow cytometry

Mixed cell suspensions from EM tissues were treated with TGFβ, E_2_ or P for 48 h prior to cell staining for flow cytometry analysis. For experiments in which intracellular cytotoxic molecules were analyzed, mixed EM CD8+T cells were examined by flow cytometry to identify CD103+and CD103− subsets as described previously^11^. Suspensions were stained for surface markers with combinations of the following antibodies: CD45-AF700, CD3-APC-Cy7, CD103-BV711 (BioLegend), CD4-PE-Cy5.5 (eBioscience, San Diego, CA), CD8-BUV395 (BD Bioscience). To detect of perforin, granzyme A (GZA) and granzyme B (GZB), following surface staining, cells were fixed and permeabilized with Cytofix/cytoperm kit (BD) according to instructions. Intracellular staining of perforin, granzyme A and B was done using combinations of the following antibodies: anti-human Perforin-PE/Dazzle, Granzyme A-AF647 (BioLegend) and Granzyme B-BV421 (BD Bioscience). Analysis was performed on BioRad ZE5 flow cytometers (BioRad) using Everest software, and data analyzed with FlowJo software version v10 (Tree Star, Inc. Ashland, OR, www.flowjo.com)**.** Expression of surface and intracellular markers was measured by the percentage of positive cells and measured median fluorescence intensity (MFI).

### Statistics

Data analysis was performed using the GraphPad Prism version 8.3.0 for Windows (GraphPad Software, San Diego, CA, www.graphpad.com). A two-sided *P* value < 0.05 was considered statistically significant. Comparison of two groups was performed with the non-parametric Wilcoxon matched-pairs signed rank test. Comparison of three or more groups was performed applying the non-parametric Friedman test with Dunn’s multiple comparisons test.

### Image generation

Figures 1 and 6 were created using GraphPad Prism version 8.3.0 for Windows (GraphPad Software, www.graphpad.com). Figures 2, 3, 4 and 5 were created using FlowJo software version v10 (www.flowjo.com) and GraphPad Prism version 8.3.0 for Windows (GraphPad Software, www.graphpad.com). Figure 7 was created using BioRender.com.

### Ethics statement

The studies were performed with Dartmouth College Institutional Review Board approval. Approval to use discarded tissues from hysterectomies was obtained from the Committee for the Protection of Human Subjects (CPHS).

## Data Availability

The datasets generated for this study are available on request to the corresponding author.
